# Investigation of cytokines, oxidative stress, metabolic, and inflammatory biomarkers after orange juice consumption by normal and overweight subjects

**DOI:** 10.3402/fnr.v59.28147

**Published:** 2015-10-20

**Authors:** Grace K. Z. S. Dourado, Thais B. Cesar

**Affiliations:** Department of Food and Nutrition, Faculty of Pharmaceutical Sciences, UNESP – Univ Estadual Paulista, Araraquara, Brazil (Faculdade de Ciências Farmacêuticas, UNESP – Univ Estadual Paulista, Departamento de Alimentos e Nutrição, Araraquara, SP 14801-902, Brazil)

**Keywords:** orange juice, overweight, biomarkers, cytokines, oxidative stress, clinical trial

## Abstract

**Background:**

Abdominal adiposity has been linked to metabolic abnormalities, including dyslipidemia, oxidative stress, and low-grade inflammation.

**Objective:**

To test the hypothesis that consumption of 100% orange juice (OJ) would improve metabolic, oxidative, and inflammatory biomarkers and cytokine levels in normal and overweight subjects with increased waist circumference.

**Design:**

Subjects were divided into two groups in accordance with their body mass index: *normal* and *overweight*. Both groups of individuals consumed 750 mL of OJ daily for 8 weeks. Body composition (weight, height, percentage of fat mass, and waist circumference); metabolic biomarkers (total cholesterol, low-density lipoprotein-cholesterol [LDL-C], high-density lipoprotein-cholesterol [HDL-C], triglycerides, glucose, insulin, HOMA-IR, and glycated hemoglobin); oxidative biomarkers (malondialdehyde and DPPH^•^); inflammatory biomarkers (high-sensitivity C-reactive protein [hsCRP]); cytokines (IL-4, IL-10, IL-12, TNF-α, and IFN-γ); and diet were evaluated before and after consumption of OJ for 8 weeks.

**Results:**

The major findings of this study were: 1) no alteration in body composition in either group; 2) improvement of the lipid profile, evidenced by a reduction in total cholesterol and LDL-C; 3) a potential stimulation of the immune response due to increase in IL-12; 4) anti-inflammatory effect as a result of a marked reduction in hsCRP; and 5) antioxidant action by the enhancement of total antioxidant capacity and the reduction of lipid peroxidation, in both normal and overweight subjects.

**Conclusions:**

OJ consumption has a positive effect on important biomarkers of health status in normal and overweight subjects, thereby supporting evidence that OJ acts as functional food and could be consumed as part of a healthy diet to prevent metabolic and chronic diseases.

Easy access to hypercaloric food items, the ingestion of high-fat and high-carbohydrate meals, as well as ever more sedentary lifestyles have contributed to the progressive increase of overweight and obesity in Western societies (1). Excess adipose tissue in the abdomen favors the induction of a cascade of events that lead to the chronic activation of the innate immune system, resulting in the development of low-grade inflammation. This condition is characterized by overproduction of proinflammatory mediators, such as circulating levels of C-reactive protein (CRP), TNF-α, and IL-1β, as well as insulin resistance and dyslipidemia, all of which are risk factors for cardiovascular diseases, metabolic syndrome, and diabetes (2). Furthermore, oxidative stress is the common factor underlying these diseases, triggering an early inflammatory response and release of adhesion molecules and cytokines (3). In contrast, the intake of various bioactive and antioxidant compounds commonly found in fruits and vegetables may counterbalance such events (4, 5).

Citrus fruits consumed as orange juice (OJ) constitute a significant source of the flavanones hesperidin and naringin in the diet (4, 6, 7). Several health benefits have been attributed to hesperidin, including anti-inflammatory, antioxidant, immunomodulatory, hypolipidemic, and anticancer effects (7). However, evidence suggests that the effects of bioactive compounds are less pronounced when ingested as dietary supplements compared with the consumption of whole fruits and juices. This is a result of synergistic interactions that occur among phytochemicals and nutrients, which can target multiple signal transduction pathways in cells (8, 9). A previous study showed that oxidative and inflammatory stress induced by high-fat and high-carbohydrate meals was prevented by the consumption of OJ in healthy subjects (10).

Consumption of 100% OJ and grapefruit was associated with better diet quality because of the increased intake of nutrients such as vitamin C, folate, and potassium; reduced risk of obesity and metabolic syndrome; as well as the lowering of blood lipids (11, 12) and lipid peroxidation (13, 14). OJ is also a moderate sugar content source, with 22 g of sugar in 250 mL, distributed as sucrose, fructose, and glucose (2:1:1). Although there are some concerns about high consumption of fructose from fruit juices, previous studies has shown that fructose intakes below 50 g/day does not represent risk for obesity or increased level of uric acid (15, 16).

Due to the paucity of clinical studies that evaluated the effect of OJ consumption on biomarkers, which are commonly altered as a consequence of excess weight and abdominal fat accumulation, we tested the hypothesis that the regular consumption of OJ would improve metabolic, oxidative, inflammatory biomarkers, and cytokines levels of overweight subjects. Therefore, we examined the OJ consumption effects on levels of serum lipids, glucose, insulin, HOMA-IR, glycated hemoglobin, high-sensitivity CRP (hsCRP), cytokines, antioxidant capacity and lipid peroxidation, quality of diet, and body composition of normal and overweight subjects.

## Material and methods

### Orange juice

One hundred percent orange juice obtained from Pera Rio oranges was kindly provided by Citrosuco, Matao, Brazil. The characteristics of the OJ, analyzed in our department, are Brix (11.5 g sucrose/100 g), titratable acidity (1.01%), ascorbic acid (26.9 mg/100 mL), total phenolics (1.66 mg/100 mL gallic acid), total flavonoids (31.6 mg rutin/mL), total carotenoids (2.00 mg β-carotene/mL), narirutin (15.0 mg/L), and hesperidin (103.3 mg/L).

### Total soluble solids and titratable acidity

Total soluble solids content (°Brix) and titratable acidity (expressed in percentage of citric acid) of samples were determined according to AOAC International (17).

### Ascorbic acid

Ascorbic acid content was determined by titration, a method based on the reduction of 2,6-dichlorophenol-indophenol by ascorbic acid, with the results expressed in milligram of ascorbic acid per 100 mL of juice (17).

### Total phenolic

Total phenolic content was determined by the Folin–Ciocalteu method (18) using gallic acid as standard. Absorbance was read at 760 nm in a Beckman Spectrophotometer DU-640. The results were expressed in milligram of gallic acid per 100 mL of juice.

### Total flavonoids

Extraction of flavonoids was perfomed using cold acetone (19). Total flavonoid content was determined by colorimetric method (20) with absorbance at 510 nm. Rutin was used as a standard to obtain a calibration curve. Results were expressed in milligram equivalent of rutin per milliliter.

### Total carotenoids

Carotenoid pigment extract was partitioned with petroleum ether. The total carotenoid content was determined by measuring the absorbance at 450 nm in a UV/V spectrophotometer using the absorption coefficient of β-carotene (A^1%^_1 cm_=2,592) (21). The total carotenoid content was expressed as mg β-carotene/mL. Carotenoid pigments were analyzed by RP-HPLC using ternary gradient of elution and Symmetry C18 column (4.6×150 mm ID., 3.5 µm) using Waters HPLC instrument (Waters Pharmaceutical Division, Milford, MA, USA). Chromatographic system was equipped with a photodiode array detector (DAD) and the mobile phase consisted of acetonitrile:methanol:ethyl acetate containing 0:05% triethylamine at a flow rate of 0.6 mL/min. A gradient was applied from 99:1:0 to 64:1:35 in 30 min and 99:1:0 in 60 min. The injection volume was 20 µL. Detection was performed at 350–550 nm. Carotenoids individually isolated in samples were identified by comparing their retention time in HPLC, by the spectra characteristics of diode array with patterns, and also by literature values.

### Flavanones hesperidin and narirutin

Extraction of flavanones was performed according to Bocco et al. (22). Orange juice (3mL) was mixed with methanol (5mL), followed by heating at 558°C for 15 min, and centrifugation at 3,150 rpm for 15 min. Supernatant was collected and the procedure was repeated twice. Methanol extracts were mixed and evaporated in a water bath at 50°C under nitrogen flow, resulting in a final volume of 10 mL with methanol. The column used in the separation of the flavanones was C18 Shimadzu Shim-pack CLC-ODS (M) (4.6×250 mm, 5 mm), and a mixture acetonitrile: water: acetic acid (21:75:4) (v/v/v) was used as mobile phase with a flow rate of 1.0 mL/min with Waters 996 DAD. Running time was 30 min and the detection was read at 280 nm. Flavanones were identified by comparing the retention times and the spectra obtained by a DAD of the samples with their respective standards (hesperidin and narirutin) between 200 and 450 nm. The results were expressed in mg/L.

### Subjects and study design

A dietary intervention clinical trial was designed to evaluate two groups of individuals in accordance with body mass index (BMI): normal weight (18.5–24.9 kg/m^2^) and overweight (≥25 kg/m^2^) (23). Fifty-five healthy individuals, aged 23–59 years, of both genders were assessed for eligibility by individual interview at Sao Paulo State University, Araraquara, Brazil. Of this sample, 25 normal weight and 25 overweight subjects were enrolled in the study because of their availability and willingness to follow the research protocol, while five subjects were excluded. Exclusion criteria were cardiovascular, kidney, and thyroid disease; diabetes; being pregnant or lactating; drug treatment for hypertension or hyperlipidemia; or be taking vitamins/minerals supplements. Twenty-one normal weight and 25 overweight participants (*n*=46) concluded the study, whereas four participants dropped out during the course of the study for professional or personal reasons and were excluded from the data analysis.

In each group, the participants daily consumed 750 mL, three cups, of 100% OJ, without extrinsic (added) sugars (24), divided into at least two intakes a day for 8 weeks. The period of intervention was based on previous studies that reported changes in serum biomarkers after OJ consumption (25, 26). Subjects were also advised to continue their habitual diets during the study. Bottles of frozen 100% OJ were delivered weekly to each participant and stored at 4°C before consumption. The researchers met with all volunteers weekly to ensure that all of them consumed 750 mL of OJ every day. None of the participants reported any difficulty in daily consumption of OJ during the period of the experiment. Body composition measures (body weight, height, fat mass, and waist circumference); metabolic biomarkers (glucose, insulin, homeostatic model assessment – insulin resistance [HOMA-IR], total cholesterol, high-density lipoprotein-cholesterol [HDL-C], low-density lipoprotein-cholesterol [LDL-C], triglycerides, glycated hemoglobin); oxidative biomarkers (malondialdehyde and DPPH^•^ radical), inflammatory biomarker (CRP); and cytokines (TNF-α, IFN-γ, IL-12, IL-4, and IL-10) were analyzed in the serum of individuals before and after 8 weeks of OJ dietary intervention.

### Body composition

Standardized weight, height, and waist circumference measurements were taken before and after the 8-week period (23). The percentage of fat mass was determined using bioelectrical impedance analysis (BIA, Biodynamics 310, Seattle, WA, USA).

### Ethics

The study was approved by the Faculty of Pharmaceutical Sciences Review Board, Sao Paulo State University for human experimentation (protocol n° 22/2009). All procedures of the study were performed in accordance with Good Clinical Practices and the World Medical Association Declaration of Helsinki. All individuals received suitable information regarding the goals of the study and the potential adverse effects and benefits of consuming 750 mL of OJ. After comprehensive information, written informed consent was requested from all individuals. It was made clear that at any time the subjects could withdraw from the study.

### Blood samples

Blood samples were drawn from each participant via the cubital vein after 12 h of fasting at baseline and at the end of the study period. Freshly drawn blood was centrifuged and the serum was stored at −80°C.

### Biomarkers

Total cholesterol, HDL-C, triglycerides, and glucose were determined by enzymatic colorimetric assay, whereas glycated hemoglobin was analyzed by immunoturbidimetric assay (Labtest Diagnostica S/A, Belo Horizonte, MG, Brazil). LDL-C was indirectly estimated (27). Insulin was measured by a chemiluminescent assay (Roche Diagnostics, Indianapolis, IN, USA). Insulin sensitivity was estimated by HOMA-IR using the equation: [fasting insulin (µU/l)×fasting glucose (mmol/l)]/22.5, and the cutoff was associated to BMI (28). Cytokines TNF-α, IFN-γ, IL-12, IL-4, and IL-10 were determined by ELISA assay (BD Pharmingen, San Jose, CA, USA). hsCRP was performed using the neflometry assay (Dade Behring Inc., Deerfield, IL, USA). Lipid peroxidation was assessed by TBARS assay measuring the amount of malondialdehyde, a secondary product of lipid peroxidation in serum (29). Total antioxidant capacity in serum was determined by DPPH^•^ radical assay (30).

### Diet assessment

Three 24-h recalls were applied for each dietary assessment at baseline and at Week 8. The participants were advised not to modify their food habits during the study. Energy, macronutrient, and micronutrient intakes were determined by the software Nut Win, version 1.5 (Federal University of Sao Paulo, Sao Paulo, Brazil).

### Statistical analysis

The minimum number of individuals (*n*) per each group was estimated adopting 5% of significance level and 80% of desired power. This calculation was based on the measurement of LDL-C in previous clinical trials performed in our lab (25, 26), which indicates that at least 22 individuals should be inserted in each group (*n*=44) in order to detect significant differences on LDL-C levels. All values were expressed as mean±standard deviation (SD), except inflammatory biomarkers and cytokines, which were expressed as mean±standard error of the mean (SEM). Repeated measures two-way ANOVA followed by post-hoc Tukey test with statistical significance set at *P*≤0.05 were used to compare the periods of OJ consumption (before and after) and groups of individuals (normal and overweight) by Sigma Stat version 3.11, Systat Software Inc., San Jose, CA, USA.

## Results and discussion

We tested the hypothesis that regular consumption of OJ could improve metabolic, oxidative, and inflammatory biomarkers and cytokines levels in overweight subjects with increased abdominal obesity. The main results found were 1) no alteration in body composition in either group; 2) improvement of the lipid profile, evidenced by a reduction in total cholesterol and LDL-C; 3) potential stimulation of the immune defense due to the increase of IL-12; 4) anti-inflammatory effect, as a result of a noticeable reduction in hsCRP levels; and 5) antioxidant action by the enhancement of total antioxidant capacity and the reduction of lipid peroxidation.

Glucose, insulin, and glycated hemoglobin levels were in accordance with reference values (31) in all subjects, but none of the normal weight, and three of the overweight subjects were insulin resistant based on HOMA-IR (28) (data not shown). Blood serum lipid panel of normal weight participants was in accordance with standard values, but overweight subjects initiated the study with total cholesterol and LDL-C at classification borderline high (32) (Table 1). In addition, overweight subjects had higher levels of triglycerides than subjects with normal weight; however, both groups were below the standard threshold values (32) (Table 1). Compared with normal weight subjects, overweight subjects had higher dietary consumption of energy (38%), carbohydrates (25%), protein (66%), lipids (36%), cholesterol (83%), and saturated fatty acids (41%) (Table 2), and, obviously, higher body weight (43%) and waist circumference (27%) (Table 1). Furthermore, the levels of total cholesterol (17%), LDL-C (33%), tryglicerides (32%), as well as TNF-α (twofold) were also higher (Table 1).

**Table 1:** Serum biomarkers and body composition of normal weight and overweight subjects after 8 weeks of dietary intervention with 750 mL of orange of juice

Subjects		Normal weight (*n*=21)		Overweight (*n*=25)	
		
Orange juice period		Before OJ	After OJ	Before OJ	After OJ
Metabolic biomarkers					
Glucose (mg/dL)		78±7^a^	77±7^a^	79±11^a^	80±11^a^
Glycated hemoglobin (%)		5.30±0.31^a^	5.30±0.30^a^	5.32±0.54^a^	5.42±0.39^a^
Insulin (µU/mL)		6.05±2.86^a^	5.28±2.83^a^	6.99±2.66^a^	7.34±2.89^a^
HOMA-IR		1.17±0.60^a^	1.01±0.58^a^	1.40±0.71^a^	1.47±0.74^a^
Triglycerides (mg/dL)		89±29^a^	84±29^a^	118±37^b^	124±36^b^
Total cholesterol (mg/dL)		173±20^a^	159±27^b^	203±40^c^	188±38^d^
LDL-C (mg/mL)		104±21^a^	93±22^b^	138±37^c^	126±37^d^
HDL-C (mg/dL)	Women	62±15^a^	57±12^a^	57±18^a^	56±17^a^
	Men	55±12^a^	48±13^a^	43±9^a^	40±7^a^
Inflammatory biomarkers*					
hsCRP (mg/dL)		0.25±0.04^a^	0.12±0.03^b^	0.18±0.02^a^	0.06±0.01^b^
Cytokines*					
IFN-γ (pg/mL)		16.5±1.8^a^	18.0±3.4^a^	16.9±1.8^a^	23.7±4.6^a^
TNF-α (pg/mL)		21.5±4.4^a^	19.1±4.0^a^	44.2±6.5^b^	40.7±7.0^b^
IL-12 (pg/mL)		7.8±1.3^a^	20.7±4.0^b^	7.4±1.0^a^	17.6±3.6^b^
IL-4 (pg/mL)		23.2±3.0^a^	19.5±3.0^a^	23.5±4.0^a^	29.7±5.8^a^
IL-10 (pg/mL)		12.5±3.3^a^	11.6±1.6^a^	12.3±2.7^a^	16.4±3.1^a^
Oxidative biomarkers					
Malondialdehyde (µM)		2.40±1.93^a^	1.55±1.20^b^	2.20±1.35^a^	1.00±0.73^b^
DPPH^•^ (%)		8.80±6.80^a^	26.0±8.64^b^	10.4±7.65^a^	26.5±8.07^b^
Body composition and age					
Age		32.1±8.6^a^		38.6±10.5^a^	
Body mass (kg)		62.1±9.4^a^	61.8±9.4^a^	88.6±11.4^b^	87.6±3.7^b^
BMI (kg/m^2^)		22.4±1.7^a^	22.3±1.7^a^	28.9±3.0^b^	28.9±2.9^b^
Body fat mass (%)		28.6±6.5^a^	29.3±4.6^a^	29.2±6.0^a^	30.4±5.3^a^
Waist circumference (cm)		77.1±7.8^a^	76.6±7.1^a^	98.1±10.6^b^	97.4±10.1^b^

Normal weight: BMI=18.5–24.9 kg/m^2^; overweight: BMI≥25 kg/m^2^ (23). Most of the results are expressed as mean±SD.

* Results are expressed as mean±SEM. Values with the same letter in a row are not significantly different, while the different letters are statisticaly significant. Repeated measures two-way ANOVA followed by post-hoc Tukey test *P*≤0.05.

**Table 2:** Energy and nutrient intakes of normal weight and overweight subjects before and after 8 weeks of dietary intervention with 750 mL of orange juice

Subjects	Normal weight (*n*=21)		Overweight (*n*=25)	
		
Orange juice period	Before OJ	After OJ	Before OJ	After OJ
Energy (kcal)	1,840±284^a^	1,886±440^a^	2,535±334^b^	2,607±334^b^
Carbohydrates (g)	234±52^a^	265±56^b^	292±79^c^	335±86^d^
Protein (g)	77±28^a^	78±22^a^	128±22^b^	121±29^b^
Lipids (g)	69±15^a^	63±20^a^	94±20^b^	82±16^c^
Cholesterol (mg)	189±61^a^	209±67^a^	346±191^b^	253±108^a^
Saturated fatty acid (g)	17±6^a^	17±6^a^	24±8^b^	20±8^ab^
Vitamin C (mg)	94±58^a^	437±40^b^	187±107^c^	451±75^b^
Folate (µg)	183±97^a^	369±70^b^	211±89^a^	432±115^d^
Calcium (mg)	691±280^a^	635±259^a^	637±243^a^	634±284^a^

Normal weight: BMI=18.5–24.9 kg/m^2^; overweight: BMI≥25 kg/m^2^ (23). Results are expressed as mean±SD. Values with the same letter in a roware not significantly different, while the different letters are statisticaly significant. Repeated measures two-way ANOVA followed by post-hoc Tukey test *P*≤0.05.

Accumulation of adipose tissue *per se* is recognized as the main contributor to insulin resistance, because of oversecretion of proinflammatory adipokines, among them, the TNF-α which can alter insulin-mediated processes, such as glucose homeostasis and lipid metabolism (33). Thus, the elevated concentration of TNF-α in overweight individuals can be associated with increased waist circumference, which predisposes them to the development of metabolic disorders, such as metabolic syndrome and type 2 diabetes (Table 1). Furthermore, higher ingestion of energy combined with an unbalanced diet in the long term may contribute to the progression of obesity, as well as the emergence of diet-related disease (1).

Regarding diet assessment (Table 2), it was observed that the total energy in diet was not altered during the 8-week intervention with OJ, indicating that this addition of energy and carbohydrate (340 kcal/day and 63 g/day of sugars) was balanced by a decrease in other macronutrients. For instance, a slight decrease in consumption of dietary lipids in normal weight subjects and a significant decrease in consumption of dietary lipids in overweight subjects diet was noted, thereby compensating the total energy intake. These changes were also noticed in previous studies where the subjects decreased the consumption of lipids and protein during the experiment (25, 34). Other data have also shown that OJ may lower the appetite, therefore accounting for reduced content of lipids and protein in the diet (35).

To evaluate the effect of less fat intake of the overweight volunteers in reducing LDL-C levels, the predictive equation of Clarke et al. (36) was applied. According to this estimative, it was found that fat from diet was responsible for a decrease of only 1.5 mg/dL LDL-C, representing only 11% of the total reduction observed (12 mg/dL). Thus, it is likely that other factors, such as bioactive compounds of OJ present chronically in the diet of these individuals, were responsible for the effect of lowering LDL-C levels. In fact, a recent study showed that changes in dietary lipids are responsible only for small and insignificant contributions to serum cholesterol levels in the blood (37). According to the study, the use of drugs to lower cholesterol has been much more pronounced in blood lipids, accounting for 46% reduction in serum cholesterol in American adults. Moreover, many studies argue that intake of citrus fruit juice (12, 38) and flavanone supplementation (39) are effective in decreasing the serum cholesterol levels in both animals and humans.

Both groups experienced an increase in vitamin C intake after OJ consumption, with a 4.6-fold increase among normal weight subjects and a twofold increase among those in the overweight group. Folate intake also had a twofold increase in both groups (Table 2). These results were expected, since OJ is a nutrient-dense beverage, and its consumption provides substantial amounts of vitamins and minerals with relatively few calories, thereby improving diet quality (40).

There were no differences in body weight, BMI, body fat mass, and waist circumference after consumption of OJ in normal weight and overweight individuals (Table 1). These data showed that body composition in both groups was not affected by the addition of 340 kcal/day provided by OJ, because of the reduction and change of the macronutrient composition in the diet. The association between the consumption of fruit juices and body weight was recently discussed in the literature (11, 41). One study that analyzed data from NHANES (2003–2006) showed that consumers of 100% OJ had lower BMI, total cholesterol, and LDL-C levels, as well as a decreased risk for obesity and metabolic syndrome (11). In addition, previous data from our group have shown that even the long-term consumption (≥12 months) of two cups of OJ daily did not modify body composition, consequently opposing the idea that plain fruit juice contributes to weight gain (38). Also, the amount of fructose offered to the volunteers in the present study (33 g/750 mL OJ) was below the threshold related to the increase of cardiometabolic risk factors (>50 g/d), and thus did not seem to contribute to weight gain or obesity (16).

There was no change in the levels of glucose, insulin, HOMA-IR values, and glycated hemoglobin among normal weight subjects after OJ treatment (Table 1). Concerning the lipid panel, there was a significant reduction in total cholesterol (−8%) and LDL-C (−11%), whereas HDL-C and triglyceride levels, in both women and men, remained unchanged. In overweight subjects, there was no change in glucose, glycated hemoglobin, HOMA-IR values, and insulin levels after OJ intake. In addition, total cholesterol and LDL-C levels were significantly reduced by 7 and 8%, respectively. HDL-C and triglyceride levels were not altered (Table 1). Similarly, in a recent study, we showed that regular consumers of OJ had lower levels of total cholesterol (−11%), LDL-C (−18%), apolipoprotein B (−12%), and LDL/HDL ratio (12%) compared with non-consumers of OJ (38). Thus, the present study highlights the lipid-lowering properties associated with OJ, as shown by the decreases in total cholesterol and LDL-C, suggesting that participants of both weight status benefited equally by OJ intake. This effect has been attributed to the flavanones, hesperidin, and naringin that modulate expression and activity of key mediators related to the control of hepatic lipid homeostasis (6, 42).

In several chronic pathological conditions, including overweight and obesity, the local inflammatory process in the adipose tissue stimulates the excessive secretion or lower levels of cytokines in circulation (43). Changes in the circulating levels of these proteins are linked to several disease states, making them valuable functional biomarkers (44). Because of the exploratory approach concerning the impact of OJ on cytokine secretion associated with excess weight, the following panel of cytokines were chosen for analysis: IL-4, IL-10, TNF-α, IFN-γ, and IL-12. The levels of IL-12 were increased approximately 2.5-fold in both normal and overweight groups. However, the cytokines IFN-γ, TNF-α, IL-4, and IL-10 did not change with OJ intake (Table 1). IL-12 is required for the development of T-helper (Th1) response and is essential for the host's defense against bacteria, viruses, and complex parasites (45, 46). Although IL-12 induces the production of IFN-γ, in the present study the levels of IFN-γ were not altered. Thus, it seems that OJ intake is able to specifically affect the secretion of IL-12, contributing to a boost in anti-infectious response, as previously observed in *ex vivo* macrophages from mice treated with hesperidin and OJ (47).

hsCRP levels among normal weight subjects were reduced by 52%, whereas among overweight individuals, the hsCRP reduction was more pronounced, by 66% (Table 1). High levels of hsCRP in blood serum (>0.3 mg/dL) have been associated with systemic inflammation, risk of coronary events (48, 49), and more recently with low-grade inflammation induced by visceral adiposity (50, 51). Participants in this experiment had baseline hsCRP between 0.1 and 0.3 mg/dL, which is associated with a moderate risk of cardiovascular diseases (48). Thus, after OJ treatment, both groups of subjects reduced the hsCRP levels to a lower risk category (≤0.1 mg/dL). The relevance of this result lies in the ability of the constituents of a natural beverage to influence the concentrations of two important predictive markers of inflammation and cardiovascular events, that is, hsCRP and LDL-C. It has been well reported that statins promote reduction in CRP and LDL-C (48, 51), but with side effects in the long term. In this sense, such results seem promising, since the modifier agent is a food source without side effects, compared with drugs. Nevertheless, more studies are necessary to support CRP-lowering action of OJ in patients with high CRP levels.

Oxidative stress is often assessed in the blood serum as TBARS, which measures the amount of malondialdehyde, a secondary product of lipid peroxidation (52). In this study, we have shown that the levels of malondialdehyde in normal and overweight subjects were within reference values (1.86–3.94 µM of malondialdehyde) for both groups of individuals (29). Furtheremore, after OJ intervention, the lipid peroxidation was significantly reduced in both normal and overweight subjects by 35 and 55%, respectively. In addition, the total antioxidant capacity test has shown a possible action of antioxidant on serum (>100%), which could be attributed to OJ consumption in both groups of individuals (Table 1). Although there was a significant reduction of malondialdehyde levels after OJ consumption in normal weight subjects (−20%), as showed previously (13), this effect was more pronounced among overweight subjects (−55%). It has been established that excessive abdominal adiposity raises the oxidative radicals that stimulates adipocytes to produce inflammatory cytokines (53), suggesting that overweight subjects were more susceptible to the antioxidant effects of OJ.

Our study has several strengths and a potential limitation. The main strength of this study is the simultaneous exploration of parameters of body composition, diet, and blood serum biomarkers in normal and overweight subjects, beyond a panel of cytokines rarely evaluated in similar studies. Moreover, our sample consisted of men and women that adhered easily to the OJ intervention, with few dropouts. The use of three dietary recalls for analysis not only showed changes in dietary consumption over time but also decreases in measurement error. The absence of control group is a limitation in this research.

We concluded that OJ may have the ability to improve metabolic, oxidative, and inflammatory biomarkers, and cytokines in both groups of individuals, normal and overweight. Most highlighted results include the potential immune-stimulating role of OJ by increasing IL-12 levels, a critical cytokine for host defense against intracellular pathogens, and absence of changes in the levels of other cytokines. Also, the ability of OJ to modulate hsCRP and LDL-C, leading to a lowering cardiovascular risk, indicates the functional role of OJ as part of a regular diet (47–49). Taken together, these outcomes suggest that the regular consumption of OJ can be a strategy to prevent diseases related to diet, since its consumption provides a unique dietary source of nutrients and bioactive compounds, which are associated with the reduction of metabolic and chronic diseases.
